# AROS: Affordance Recognition with One-Shot Human Stances

**DOI:** 10.3389/frobt.2023.1076780

**Published:** 2023-05-02

**Authors:** Abel Pacheco-Ortega, Walterio Mayol-Cuevas

**Affiliations:** ^1^ Visual Information Lab, Department of Computer Science, University of Bristol, Bristol, United Kingdom; ^2^ Amazon.com, Seattle, WA, United States

**Keywords:** affordance detection, scene understanding, human interactions, visual perception, affordances

## Abstract

We present Affordance Recognition with One-Shot Human Stances (AROS), a one-shot learning approach that uses an explicit representation of interactions between highly articulated human poses and 3D scenes. The approach is one-shot since it does not require iterative training or retraining to add new affordance instances. Furthermore, only one or a small handful of examples of the target pose are needed to describe the interactions. Given a 3D mesh of a previously unseen scene, we can predict affordance locations that support the interactions and generate corresponding articulated 3D human bodies around them. We evaluate the performance of our approach on three public datasets of scanned real environments with varied degrees of noise. Through rigorous statistical analysis of crowdsourced evaluations, our results show that our one-shot approach is preferred up to 80% of the time over data-intensive baselines.

## 1 Introduction

Vision evolved to make inferences in a 3D world, and one of the most important assessments we can make is what can be done where. Detecting such environmental affordances allows the identification of locations that support actions, such as stand-able, walk-able, place-able, and sit-able. Human affordance detection is not only important in scene analysis and scene understanding but also potentially beneficial in object detection and labeling (*via* how objects can be used) and can eventually be useful for scene generation as well.

Recent approaches have worked toward providing such key competency to artificial systems *via* iterative methods, such as deep learning (Zhang et al., 2020a; Bochkovskiy et al., 2020; Carion et al., 2020; Du et al., 2020; Nekrasov et al., 2021). The effectiveness of these data-driven efforts is highly dependent on the number of classes, the number of examples per class, and their diversity. Usually, a dataset consists of thousands of examples, and the training process requires a significant amount of hand tuning and computing of resources. When a new category needs to be added, further sufficient samples need to be provided and training remade. The appeal for one-shot training methods is clear.

**FIGURE 1:**
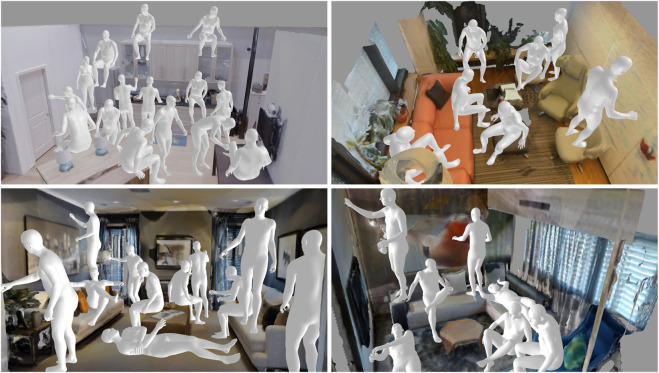
AROS is capable of detecting human–scene interactions with one-shot learning. Given a scene, our approach can detect locations that support interactions and generate the interacting human body in a natural and plausible way. Images show examples of detected sit-able, reach-able, lie-able, and stand-able locations.

Often, human pose-in-scene detection is conflated with object detection or other semantic scene recognition, for example, training to detect sit-able locations through chair recognition, while this is a flawed approach for general action-scene understanding, first, since people can recognize numerous non-chair locations where they can sit, e.g., on tables or cabinets (Figure 1). Second, an object-driven approach may fail to consider that affordance detection depends on the object pose and its surroundings—it should not detect a chair as sit-able if it is upside-down or if an object is over it. Finally, object detectors alone may struggle to perceive a potentially sit-able place if a particular object example was not covered during training.

To address these limitations, Affordance Recognition with One-shot Human Stances (AROS) uses a direct representation of human-scene affordances. It extracts an explainable geometrical description by analyzing proximity zones and clearance space between interacting entities. The approach allows training from one or very few data samples per affordance and is capable of handling noisy scene data as provided by real visual sensors, such as RGBD and stereo cameras.

In summary, our contributions are as follows: 1) we propose a one-shot learning geometric-driven affordance descriptor that captures both proximity zones and clearance space around human–pose interactions. 2) We set a statistical framework that relies on both central tendency statistics and a statistical inference to evaluate the performance of the compared approaches. The tests show that our approach generates natural and physically plausible human–scene interactions with better performance than intensively trained state-of-the-art methods. 3) Our approach demonstrates control on the kind of human–scene interaction sought, which permits exploring scenes with a concatenation of affordances.

## 2 Related work

Following Gibson’s suggestion that affordances are what we perceive when looking at scenes or objects (Gibson, 1977), the perception of human affordances with computational approaches has been extensively explored over the years. Before the popularity of data-intensive approaches, Gupta et al. (2011) employed an environment geometric estimation and a voxelized discretization of four human poses to measure the environment affordance capabilities. This human pose method was employed by Fouhey et al. (2015) to automatically generate thousands of labeled RGB frames from the NYUv2 dataset (Silberman et al., 2012) for training a neural network and a set of local discriminative templates that permits the detection of four human affordances. A related approach was explored by Roy and Todorovic (2016), where detection was performed for five different human affordances through a pipeline of CNNs that includes the extraction of mid-level cues trained on the NYUv2 dataset (Silberman et al., 2012). Luddecke and Worgotter (2017) implemented a residual neural network for detecting 15 human affordances and trained using a look-up table that assigns affordances to object parts on the ADE20K dataset (Zhou et al., 2017).

Another research line has been the creation of action maps. Savva et al. (2014) generated affordance maps by learning relations between human poses and geometries in recorded human actions. Piyathilaka and Kodagoda (2015) used human skeleton models positioned in different locations in an environment to measure geometrical features and determine the support required. In Rhinehart and Kitani (2016), egocentric videos as well as scenes, objects, and actions classifiers were used to build up the action maps.

There have been efforts to use functional reasoning for describing the purpose of elements in the environment that helped define them. Grabner et al. (2011) designed a geometric detector for sit-able objects, such as chairs, while further explorations performed by Zhu et al. (2016) and Wu et al. (2020) included physics engines to ponder constrains, such as collision, inertia friction, and gravity.

An important line of research is focused on generating human–environment interactions, representative of affordances detected in the environment. Wang et al. (2017) proposed an affordance predictor and a 2D human interaction generator trained on more than 20K images extracted from sitcoms with and without humans interacting with the environment. Li et al. (2019) extended this work by developing a 3D human pose synthesizer that learns on the same dataset of images but generates human interactions into input scenes that are represented as RGB, RGBD, or depth images. Jiang et al. (2016) exploited the spatial correlation between elements and human interactions on RGBD images to generate human interactions and improve object labeling. These methods use human skeletons for representing body–environment configurations, which reduces their representativeness since contacts, collisions, and naturalness of the interactions cannot be evaluated in a reliable manner.

In further studies, Ruiz and Mayol-Cuevas (2020) developed a geometric interaction descriptor for non-articulated, rigid object shapes. Given a 3D environment, the method demonstrated good generalization on detecting physically feasible object–environment configurations. In the SMPL-X human body representation (Pavlakos et al., 2019), Zhang et al. (2020c) presented a context-aware human body generator that learned the distribution of 3D human poses conditioned to the scene depth and semantics via recordings from the PROX (Hassan et al., 2019) dataset. In a follow-up effort, Zhang et al. (2020b) developed a purely geometrical approach to model human–scene interactions by explicitly encoding the proximity between the body and the environment, thus only using a mesh as input. Training CNNs and related data-driven methods require the use of most, if not all, of the labeled dataset; e.g., in PROX (Hassan et al., 2019), there are 100K image frames.

## 3 AROS

Detecting human affordances in an environment is to find locations capable of supporting a given interaction between a human body and the environment. For example, the study of finding “suitable to sit” locations identifies all those places where a human can sit, which can include a range of object “classes” (sofa, bed, chair, table, etc.). Our method is motivated to develop a descriptor that characterizes such general interactions without requiring object classes by using two key components and that is lightweight in terms of data requirements while outperforming alternative baselines.

**FIGURE 2:**
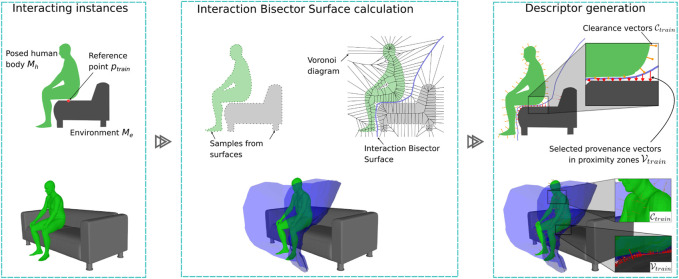
2D and 3D illustrations of our one-shot training pipeline. (Left) Posed human body *M*_*h*_ interacting with an environment *M*_*e*_ on a reference point *p*_*train*_. (Center) Only during training, we calculate the Voronoi diagram with sample points from both the environment and body surfaces to generate an IBS. (Right) We use the IBS to characterize the proximity zones and the surrounding space with provenance and clearance vectors. A weighted sample of these provenance and clearance vectors, $V_{\mathrm{train}}$ and $C_{\mathrm{train}}$ , respectively, results in good generalization of the interaction.

These two components weigh the extraction of characteristics from areas with high (contact) and low (clearance) physical proximity between the entities in interaction (Figure 2).

Importantly, the representation allows one-shot training per affordance, which is desirable to improve training scalability. Furthermore, our approach is capable of describing and detecting interactions between noisy data representations as obtained from visual depth sensors and highly articulated human poses.

### 3.1 A spatial descriptor for spatial interactions

We are inspired by recent methods that have revisited geometric features, such as the bisector surface for scene–object indexing (Zhao et al., 2014) and affordance detection (Ruiz and Mayol-Cuevas, 2020). Initiating from a spatial representation makes sense if it helps reduce data training needs and simplify explanations—as long as it can outperform data-intensive approaches. Our affordance descriptor expands on the Interaction Bisector Surface (IBS) (Zhao et al., 2014), an approximation of the well-known Bisector Surface (BS) (Peternell, 2000). Given two surfaces $S_{1},S_{2}\in R^{3}$ , the BS is the set of sphere centers that touch both surfaces at one point each. Due to its stability and geometrical characteristics, the IBS has been used in context retrieval, interaction classification, and functionality analysis (Zhao et al., 2014; Hu et al., 2015; Hu et al., 2016; Zhao et al., 2016; Zhao et al., 2017; Ruiz and Mayol-Cuevas, 2020). Our approach expands on these ideas and is geometrically intuitive and straightforward. It explicitly captures areas that are important to be in scene-contact and those that are not. Importantly, we show how this approach can be generalized from just one or a small number of samples to a large unseen number of scenes.

Our one-shot training process represents interactions by 3-tuples (*M*_*h*_, *M*_*e*_, and *p*_*train*_), where *M*_*h*_ is a posed human-body mesh, *M*_*e*_ is an environment mesh, and *p*_*train*_ is the reference point on *M*_*e*_ that supports the interaction. Let *P*_*h*_ and *P*_*e*_ be the sets of samples on *M*_*h*_ and *M*_*e*_, respectively, their IBS I is defined as

$$I=\left\{p|\underset{p_{h}^{\prime }\in P_{h}}{\min}\|p-p^{\prime }\|=\underset{p_{e}^{\prime }\in P_{e}}{\min}\|p-p^{\prime }\|\right\}$$ (1)

We use the Voronoi diagram D generated with *P*_*h*_ and *P*_*e*_ to produce I . By construction, every ridge in D is equidistant to the couple of points that defined it. Then, I is composed of ridges in D generated because of points from both *P*_*h*_ and *P*_*e*_. An IBS can reach infinity, but we limit I by clipping it with the bounding sphere of *M*_*h*_ with tolerance *ibs*_*rf*_.

The number and distribution of samples in *P*_*h*_ and *P*_*e*_ are crucial for a well-constructed discrete IBS. A low rate of sampled points degenerates on an IBS that pierces the boundaries of *M*_*h*_ or *M*_*e*_. A higher density is critical in those zones where the proximity is high. To populate *P*_*h*_ and *P*_*e*_, we first use a Poisson-disc sampling strategy (Yuksel, 2015) to generate *ibs*_*ini*_ evenly distributed samples on each mesh surface. Then, we perform a *counter-part sampling* that increases the number of samples in *P*_*e*_ by including the closest points on *M*_*e*_ to elements in *P*_*h*_, and similarly, we incorporate in *P*_*h*_ the closest point on *M*_*h*_ to samples in *P*_*e*_. We perform the *counter-part sampling* strategy *ibs*_*cs*_ times to generate a new I . However, we observed that for intricate human–scene poses, convergence to an IBS without mesh piercing is challenging. If the IBS is penetrating the scene, we perform a *collision-point sampling* strategy. This adds as sampling points, a sub-sample of points where collisions happen and their counter-part points (body or environment). We then simply recompute the IBS and repeat the *counter-part sampling* and *collision-point sampling* strategies until we find a candidate I that does not collide with *M*_*h*_ or *M*_*e*_. This is a straightforward process that can be implemented efficiently.

To capture the regions of interaction proximity on our enhanced IBS as mentioned above, we use the notion of provenance vectors (Ruiz and Mayol-Cuevas, 2020). The *provenance vectors* of an interaction start from any point on I and finish on *M*_*e*_. Formally,

$$V_{p}=\left\{\left(a,\vec{v}\right)|a\in I,\;\vec{v}=\underset{e\in M_{e}}{\mathrm{arg min}}\|e-a\|-a\right\}$$ (2)

where *a* is the stating point of the delta vector $\vec{v}$ to the nearest point on *M*_*e*_.

*Provenance vectors* inform about the direction and distance of the interaction; the smaller the $\left|\vec{v}\right|$ , the more important it is in the description. Let $V_{p}^{\prime }\subset V_{p}$ be the subset of *provenance vectors* that finish on any point in *P*_*e*_, and we perform a weighted randomized selection sampling of elements from $V_{p}^{\prime }$ with the allocation of weights as follows:

$$w_{i}=1-\frac{\left|{\vec{v}}_{i}|-|{\vec{v}}_{\min}\right|}{\left|{\vec{v}}_{\max}|-|{\vec{v}}_{\min}\right|},\;i=1,\;2,\;\ldots ,\;|P_{e}|$$ (3)

where $\left|{\vec{v}}_{\max}\right|$ and $\left|{\vec{v}}_{\min}\right|$ are the norms of the biggest and smallest vectors in $V_{p}^{\prime }$ , respectively. The selected *provenance vectors* $V_{\mathrm{train}}$ integrate to our affordance descriptor with an adjustment to normalize their positions, with the defined reference point *p*_*train*_ as follows:

$$V_{\mathrm{train}}=\left\{\left(a_{i}^{\prime },{\vec{v}}_{i}\right)|a_{i}^{\prime }=a_{i}-p_{\mathrm{train}},\;i=1,\;2,\;\ldots ,\;num_{pv}\right\}$$ (4)

where *num*_*pv*_ is the number of samples from $V_{p}^{\prime }$ to integrate. The *provenance vectors* alone, however, are insufficient to work successfully on highly articulated objects, such as human poses. They are unable to capture the whole nature of the interaction. We expand this concept by taking a more comprehensive description that considers both areas of the IBS, those that are proximal to surfaces and those that are not.

We include a set of vectors into our descriptor to define the clearance space necessary for performing the given interaction. Given *S*_*h*_, an evenly sampled set of *num*_*cv*_ points on *M*_*h*_, the *clearance vectors* that integrate to our descriptor $C_{\mathrm{train}}$ on the interaction are defined as follows:

$$C_{\mathrm{train}}=\left\{\left(s_{j}^{\prime },{\vec{c}}_{j}\right)|s_{j}^{\prime }=s_{j}-p_{\mathrm{train}},\;s_{j}\in S_{h},\;{\vec{c}}_{j}=\psi \left(s_{j},\;{\hat{n}}_{j},\;I\right)\right\}$$ (5)

$$\psi \left(s_{j}^{\prime },{\hat{n}}_{j},I\right)=\left\{\begin{array}{cc} d_{\max}\cdot {\hat{n}}_{j}\;\;\;\; & \text{if }\varphi \left(s_{j},\;{\hat{n}}_{j},\;I\right)>d_{\max}\\ \varphi \left(s_{j},{\hat{n}}_{j},I\right)\cdot {\hat{n}}_{j}\;\;\;\; & \text{otherwise} \end{array}\right.$$ (6)

where *p*_*train*_ is the defined reference point, ${\hat{n}}_{i}$ is the unit surface normal vector on sample *s*_*j*_, *d*_*max*_ is the maximum norm of any ${\vec{c}}_{j}$ , and $\varphi \left(s_{j},\;{\hat{n}}_{j},\;I\right)$ is the distance traveled by a ray with origin *s*_*j*_ and direction ${\hat{n}}_{i}$ until collision with I .

Formally, our affordance descriptor, AROS, is defined as

$$f:\left(M_{h},M_{e},p_{\mathrm{train}}\right)\to \left(V_{\mathrm{train}},C_{\mathrm{train}},{\hat{n}}_{\mathrm{train}}\right)$$ (7)

where ${\hat{n}}_{\mathrm{train}}$ is the unit normal vector on *M*_*e*_ at *p*_*train*_. We calculate ${\hat{n}}_{\mathrm{train}}$ for speeding up the detection process.

### 3.2 Human affordance detection

Let $A=\left(V_{\mathrm{train}},C_{\mathrm{train}},{\hat{n}}_{\mathrm{train}}\right)$ be an affordance descriptor; we define its rigid transformation with $\tau \in R^{3}$ being a translation vector and *ϕ* being the rotation around *z* defined by *R*_*ϕ*_.

Given a point *p*_*test*_ on an environment mesh *M*_*test*_ and its unit surface normal vector ${\hat{n}}_{\mathrm{test}}$ , we determine that such a location supports a trained interaction A if we can find that (1) has a small angle difference between ${\hat{n}}_{\mathrm{test}}$ and ${\hat{n}}_{\mathrm{train}}$ , (2) once translated to *p*_*test*_ and oriented with *ϕ*_*test*_, there is a correct alignment of $V_{\phi \tau }^{A}$ , and (3) a gated number of the $C_{\phi \tau }^{A}$ is in collision with *M*_*test*_.

A significant angle difference between ${\hat{n}}_{\mathrm{test}}$ and ${\hat{n}}_{\mathrm{train}}$ permits to short-cut the test and reject *p*_*test*_ with reference to A . We establish $\rho_{\vec{n}}$ as the decision threshold for the angle difference. $\rho_{\vec{n}}$ is adjustable based on the level of mesh noise.

If we observe a normal match between ${\hat{p}}_{\mathrm{train}}$ and *p*_*test*_ vectors, we perform transformations over the interaction descriptor A with *τ* = *p*_*test*_ and *n*_*ϕ*_ different *ϕ* = *ϕ*_*test*_ values within [0, 2*π*]. Hence, per each 3-tuple $\left(V_{\phi \tau }^{A},\;C_{\phi \tau }^{A},\;{\hat{n}}_{\mathrm{train}}\right)$ calculated, we generated a set of rays *R*_*pv*_ defined as follows:

$$R_{pv}=\left\{\left(a_{i}^{\prime \prime },{\hat{\nu }}_{i}\right)\;|\;{\hat{\nu }}_{i}=\frac{\vec{v}i}{\left\|\vec{v}i\right\|},\;\left(a_{i}^{\prime \prime },{\vec{v}}_{i}\right)\in V_{\phi \tau }^{A}\right\}$$ (8)

where $a_{i}^{\prime \prime }$ is the starting point and ${\hat{\nu }}_{i}\in R^{3}$ is the direction of each ray. We extend each ray in *R*_*pv*_ by $\epsilon_{i}^{pv}$ until collision with *M*_*test*_ as

$$\left(a^{\prime \prime }+\epsilon_{i}^{pv}\cdot {\hat{\nu }}_{i}\right)\in M_{\mathrm{test}},\;\;i=1,2,\ldots ,num_{pv}$$ (9)

and compare with the magnitude of each correspondent provenance vector in $V_{\phi \tau }^{A}$ . When any element in *R*_*pv*_ extends further than a predetermined limit *max*_*long*_, the collision with the environment is classified as non-colliding. We calculate the alignment score *κ* as a sum difference between extended rays and *provenance vectors* with

$$\kappa =\underset{\forall i|\epsilon_{i}^{pv}\leq max_{\mathrm{long}}}{\sum }|\epsilon_{i}^{pv}-{\vec{v}}_{i}|$$ (10)

The bigger the *κ* value, the less the support for the interaction on the *p*_*test*_. We experimentally determine interaction-wise thresholds for the sum of differences *max*_*κ*_ and the number of missing ray collisions *max*_*missings*_ that permits us to score the affordance capabilities on *p*_*test*_.

*Clearance vectors* are meant to fast-detect collision configurations by ray–mesh intersection calculation. Similar to *provenance vectors*, we generate a set of rays *R*_*cv*_, whose origins and directions are determined by $C_{\phi \tau }^{A}$ . We extend rays in *R*_*cv*_ until collision with the environment and calculate its extension $\epsilon_{j}^{cv}$ . Extended rays with $\epsilon_{j}^{cv}\leq \|{\vec{c}}_{j}\|$ are considered as possible collisions. In practice, we also track an interaction-wise threshold to refuse affordance due to collisions *max*_*collisions*_.

**FIGURE 3:**
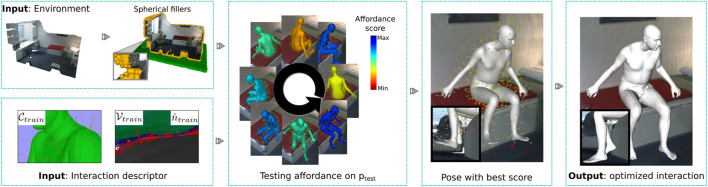
Approach for detecting human affordances. To mitigate 3D scan noise, the scene is augmented with spherical fillers for detecting collisions and SDF values. Our method detects if a test point in the environment can support an interaction by translating the descriptor to the test position over different orientations and measuring its alignment and collision rate. Then, the best-scored configuration is optimized to generate a more natural and physically plausible interaction with the environment.

A sparse distribution of clearance vectors on bi-dimensional noisy meshes in a 3D space results in collisions that are not detected by *clearance vectors*. To improve, we enhance scenes with a set of *spherical fillers* that pad the scene (see Figure 3). More details are provided in Supplementary Material.

#### 3.2.1 Pose optimization

After a positive detection, we generate the body mesh representation used in training at the testing location. This generally has low levels of contact with the unseen environment. These gaps are because our descriptor based its construction on the bisector surface between the interacting entities. We can eliminate the gap by translating the body until it touches the environment. However, this naïve method generates configurations that visually lack naturalness, Figure 3 (Pose with best score).

Every human–environment configuration trained has an associated 3D human SMPL-X characterization that we keep and use to optimize the human pose as in the work of Zhang S. et al. (2020b) with the *AdvOptim* loss function, using the SDF values that have been pre-calculated in each scene with a grid of 256 × 256 × 256 positions.

**FIGURE 4:**
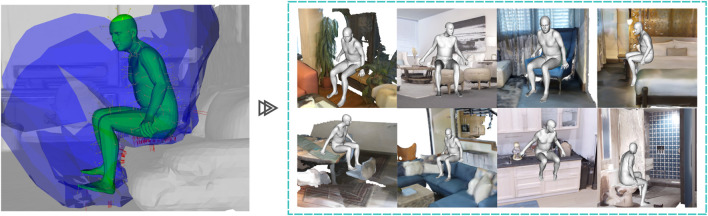
Our one-shot learning approach generalizes well on affordance detection. Only one example of an interaction is used to generate an AROS descriptor that generalizes well for the detection of affordances over previously unseen environments.

Overall, we train a human interaction by generating its AROS descriptor from a single example, keeping the associated SMPL-X parameters of the body pose and defining the contact regions that the body has with the environment. After a positive detection with AROS, we use the associated SMPL-X body parameters and its contact regions to close the environment–body gap and generate a more natural body pose, as shown in Figure 3 (ouput). Our approach generalizes well on the description of interaction and generates natural and physically plausible body–environment configurations over novel environments with just one example for training (see Figure 4).

## 4 Experiments

We conduct experiments in various environment configurations to examine the effectiveness and usefulness of the affordance recognition performed by AROS. Our experiments include several perceptual studies, as well as a *physical plausibility* evaluation of the body–environment configurations generated.

Datasets: The PROX dataset (Hassan et al., 2019) includes data from 20 recordings of subjects interacting within 12 scanned indoor environments. An SMPL-X body model (Pavlakos et al., 2019) is used to characterize the shape and pose of humans within each frame in recordings. Following the setup in the work of Zhang S. et al. (2020b), we use the rooms MPH16, MPH1Library, N0SittingBooth, and N3OpenArea for testing purposes and training on data from other PROX scenes. We also perform evaluations on seven scanned scenes from the MP3D dataset (Chang et al., 2017) and five scenes from the Replica dataset (Straub et al., 2019). We calculate the *spherical fillers* and SDF values of all 3D scanned environments.

Training: We manually select 23 frames in which subjects interact in one of the following ways: sitting, standing, lying down, walking, or reaching. From these selected human–scene interactions, we generate the AROS descriptors and retain the SMPL-X parameters associated with human poses.

To generate the IBS associated with each trained interaction, we use an initial sampling set of *ibs*_*ini*_ = 400 on each surface, execute the *counter-part sampling* strategy *ibs*_*cs*_ = 4 times, and crop the generated IBS I with *ibs*_*rf*_ = 1.2. The AROS descriptors are a compound of *num*_*pv*_ = 512 *provenance vectors* and *num*_*cv*_ = 256 *clearance vectors* that extend up to *d*_*max*_ = 5 [*cm*] each.

The interaction-wise thresholds *max*_*κ*_, *max*_*missings*_, and *max*_*collisions*_ are established experimentally, and *max*_*long*_ is 1.2 times the radius of the sphere used to crop I . We use a moderate angle difference threshold of $\rho_{\vec{n}}=\pi /3$ , in *n*_*ϕ*_ = 8 different directions.

With 512 provenance vectors $V_{\mathrm{train}}$ and 256 clearance vectors $C_{\mathrm{train}}$ , the AROS descriptor characterizes an interaction with less than 40 KB, including the SMPL-X parameters.

Baselines: We compare our approach with the state-of-the-art PLACE (Zhang et al., 2020b) and POSA (contact only) (Hassan et al., 2021). PLACE is a pure scene-centric method that only requires a reference point on a scanned environment to generate a human body performing around it. However, PLACE does not have control over the type of interaction detected/generated. We used naive and optimized versions of this approach in experiments (PLACE, PLACE SimOptim, and PLACE AdvOptim). POSA is a human-centric approach that, given a posed human body mesh, calculates the zones on the body where contact with the scene may occur and uses this feature map to place the body in the environment. We encourage a fair comparison by evaluating the naive and optimized POSA versions that consider only contact information and excludes semantic information (POSA and POSA optimized). In our studies, POSA was executed with the same human shapes and poses used to train AROS.

### 4.1 Physical plausibility

We evaluate the physical plausibility of the compared approaches mainly by following the work of Zhang et al. (2020b) and Zhang et al. (2020c). Given the SDF values of a scene and a body mesh generated, 1) the *contact score* is assigned to 1 if any mesh vertex has a negative SDF value and is evaluated as 0, otherwise, 2) the *non-collision score* is the ratio of vertices with a positive SDF value, and 3) in order to measure the severity of the body–environment collision on positive contact, we include the *collision-depth score*, which averages the depth of the collisions between the scene and the generated body mesh.

#### 4.1.1 Ablation study

We evaluate the influence of *clearance vectors*, spherical fillers, and different optimizers on the PROX dataset. Three different optimization procedures are evaluated. The *downward* optimizer translates the generated body downward (-Z direction) until it comes in contact with the environment. The ICP optimizer uses the well-known Interactive Closest Point algorithm to align the body vertices with the environment mesh. The *AdvOptim* optimizer is described in Section 3.2.1.

Table 1 shows that models without *clearance vectors* have the highest collision-depth scores on models with the same optimizer. AROS models present a reduction in contact and collision-depth scores in all cases that consider *clearance vectors* in their descriptors to avoid collision with the environment. Spherical fillers have a significant influence on avoiding collisions, producing the best scores in all metrics per optimizer. The ICP optimizer closes the body–environment gaps but drastically reduces the performance on both collision scores, while the *AdvOptim* and *downward* optimizers keep a trade-off between collision and contact. The best performance is achieved with affordance descriptors composed of *provenance* and *clearance vectors*, tested in scanned environments enhanced with *spherical fillers*, and where interactions are optimized with the *AdvOptim* optimizer.

**TABLE 1:** Ablation study evaluation scores (^*↑*^: benefit; ^*↓*^: cost). The best trade-off between scores per optimizer are in boldface.

Descriptor integrated by	Spherical filler	Optimizer	Non-collision^*↑*^	Contact^*↑*^	Collision-depth^*↓*^
$V_{\mathrm{train}}$	No	w/o	0.9348	0.7998	1.4132
$V_{\mathrm{train}},C_{\mathrm{train}}$	No		0.9504	0.6901	0.6757
$V_{\mathrm{train}},C_{\mathrm{train}}$	Yes		**0.9623**	**0.5448**	**0.1573**
$V_{\mathrm{train}}$	No	ICP^a^	0.5820	1.0000	7.3770
$V_{\mathrm{train}},C_{\mathrm{train}}$	No		0.5775	1.0000	7.2180
$V_{\mathrm{train}},C_{\mathrm{train}}$	Yes		**0.6299**	1.0000	**6.2665**
$V_{\mathrm{train}}$	No	Downward	0.9271	0.9377	1.4380
$V_{\mathrm{train}},C_{\mathrm{train}}$	No		0.9496	0.9036	0.7089
$V_{\mathrm{train}},C_{\mathrm{train}}$	Yes		**0.9641**	**0.8603**	**0.1807**
$V_{\mathrm{train}}$	No	AdvOptim	0.9552	0.9638	2.0249
$V_{\mathrm{train}},C_{\mathrm{train}}$	No		0.9717	0.9508	1.2325
$V_{\mathrm{train}},C_{\mathrm{train}}$	Yes		**0.9818**	**0.9403**	**0.6341**

a ICP stands for the Iterative Closest Point.

#### 4.1.2 Comparison with the state of the art

We generated 1300 interacting bodies per model in each of the 16 scenes and reported the averages of calculated non-collision, contact, and collision-depth scores. The results are shown in Table 2. In all datasets, interacting bodies generated using our approach provided a good trade-off with high non-collision but low contact and collision-depth scores.

**TABLE 2:** Physical plausibility: Non-collision, contact, and collision-depth scores (^*↑*^: benefit; ^*↓*^: cost) before and after optimization. The best results are in boldface.

		Non-collision^*↑*^			Contact^*↑*^			Collision-depth^*↓*^		
Model	Optimizer	PROX	MP3D	Replica	PROX	MP3D	Replica	PROX	MP3D	Replica
PLACE	w/o	0.9207	0.9625	0.9554	0.9125	0.5116	0.8115	1.6285	0.8958	1.2031
PLACE	SimOptim	0.9253	0.9628	0.9562	0.9263	0.5910	0.8571	1.8169	1.0960	1.5485
PLACE	AdvOptim	0.9665	0.9798	0.9659	0.9725	0.5810	0.9931	1.6327	1.1346	1.6145
POSA	w/o	**0.9820**	0.9792	0.9814	0.9396	0.9526	0.9888	1.1252	1.5416	2.0620
POSA	Optimized	0.9753	0.9725	0.9765	**0.9927**	**0.9988**	**0.9963**	1.5343	2.0063	2.4518
AROS	w/o	0.9615	**0.9853**	**0.9931**	0.5654	0.3287	0.4860	**0.1648**	**0.1326**	**0.2096**
AROS	AdvOptim	0.9816	**0.9853**	0.9883	0.9363	0.6213	0.8682	0.6330	0.8716	0.8615

### 4.2 Perception of naturalness

We use Amazon Mechanical Turk to compare and evaluate the naturalness of body–environment configurations generated by our approach and baselines. We used only the best version of the compared methods (with optimizer). Each scene in our test set was used equally to select 162 locations around which the compared approaches generate human interactions. MTurk judges observed all human–environment pairs generated through dynamic views, allowing us to showcase them from different perspectives. Each judge performed 11 randomly selected assessments, without repetition, that included two control questions to detect and exclude untrustworthy evaluators. Three different judges accomplished each of the evaluations. Our perceptual experiments include individual and comparison studies for each comparison carried out.

In our side-by-side comparison studies, interactions detected/generated from two approaches are exposed simultaneously. Then, MTurkers were asked to respond to the question “Which example is more natural?” by direct selection.

We used the same set of interactions for individual evaluation studies, where judges rated every individual human–scene interaction by responding to “The human is interacting very naturally with the scene. What is your opinion?” with a 5-point Likert scale according to its agreement level: 1) strongly disagree, 2) disagree, 3) neither disagree nor agree, 4) agree, and 5) strongly agree.

#### 4.2.1 Randomly selected test locations

The first group of studies compares human–scene configurations generated at randomly selected locations. On the side-by-side comparison study that contrasts AROS with PLACE, our approach was selected as more natural in 60.7% of all assessments. Compared to POSA, ours is selected in 72.6% of all tests performed. The results per dataset are shown in Table 4 (% preferences in random locations).

Individual evaluation studies also suggest that AROS produced more natural interactions (see Table 3). The mean and standard deviations of these scores obtained by the judges to PLACE are 3.23 ± 1.35 in comparison with AROS, 3.39 ± 1.25, while in the second study, these statistics obtained by POSA were 2.79 ± 1.18 in contrast with AROS, 3.20 ± 1.18. Evaluation scores of AROS have a larger mean and a narrower standard deviation compared to baselines. However, these descriptive statistics must be cautiously used as evidence to determine a performance difference because it assumes that the distribution of scores approximately resembles a normal distribution and that the ordinal variable was perceived as numerically equidistant by judges. Regrettably, Shapiro–Wilk tests (Shapiro and Wilk, 1965) performed on data show that the score distributions depart from normality in both evaluation studies, PLACE/AROS and POSA/AROS with *p* < 0.01.

**TABLE 3:** Cross-tabulation data of individual evaluation studies on randomly selected locations. The best are in boldface.

Individual evaluation study	Model		1. Stronglydisagree	2. Disagree	3. Neither	4. Agree	5. Strongly agree
PLACE *vs*. AROS	PLACE	Observed frequency	68	98	70	153	97
		% within model	14.0	20.2	14.4	31.5	19.9
	AROS	Observed frequency	**43**	**98**	64	**187**	**94**
		% within model	**8.8**	**20.2**	13.2	**38.5**	**19.3**
POSA *vs*. AROS	POSA	Observed frequency	64	173	89	123	37
		% within model	13.2	35.6	18.3	25.3	7.6
	AROS	Observed frequency	**29**	**136**	85	**179**	**57**
		% within model	**6.0**	**28.0**	17.5	**36.8**	**11.7**

Based on this, we performed a chi-square test of homogeneity (Franke et al., 2012) with a significance level *α* = 0.05, to determine if the distributions of evaluation scores are statistically similar. If we observe significance, the level of association between the approach and the distribution of the scores was determined by calculating Cramer’s V value (*V*) (Cramer, 1946).

In this first set of randomly selected locations, data from the PLACE/AROS evaluation suggest that there is no statistically significant difference between score distributions ( $\chi_{\left(4\right)}^{2}=9.34$ , *p* = 0.053). A larger sample size may be necessary to observe statistical significance; however, this will be of negligible size effect. Nevertheless, data from the POSA/AROS evaluation study showed that our approach performs better than POSA ( $\chi_{\left(4\right)}^{2}=32.33$ , *p* < 0.001) with a medium level of association (*V* = 0.1823).

#### 4.2.2 Challenging test locations

**FIGURE 5:**
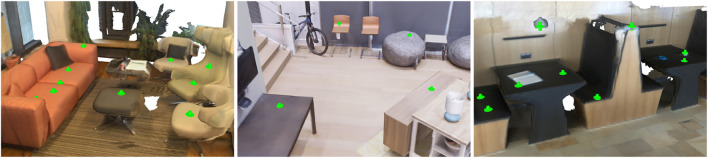
Selected by a golden annotator, green spots correspond to examples of meaningful, challenging locations for affordance detection.

A random sampling strategy is insufficient to fully evaluate the performance of pose affordances, since what matters for such methods is how they perform under realistic albeit challenging specific scene locations. For example, a test can be oversimplified and inadequate for evaluations if the sampled scene has relatively large empty spaces where only the floor or a big plane surface surrounds the test locations. Therefore, we crowdsource the evaluations in a new set of more realistic locations provided by a golden annotator (none of the authors) tasked with identifying areas of interest for human interactions (Figure 5). These locations are available for comparison as part of our dataset (https://abelpaor.github.io/AROS/).

The results of the side-by-side comparison studies confirm that in 60.6% of the comparisons with PLACE, AROS was considered more natural overall. Compared to POSA, AROS was marked with better performance in 76.1% of all evaluations with a notorious difference in MP3D locations, where AROS was evaluated to be more natural in 80.2% of the assessments. The results per dataset are shown in Table 4 (% preferences in challenging locations).

**TABLE 4:** MTurk side-by-side studies results in random and challenging locations. The best are in boldface.

		% preferences in random locations			% preferences in challenging locations		
Side-by-side comparison study	Model	MP3D	PROX	Replica	MP3D	PROX	Replica
PLACE *vs*. AROS	PLACE	39.5	32.7	45.7	38.9	30.9	36.4
	AROS	**60.5**	**67.3**	**54.3**	**61.1**	**69.1**	**63.6**
POSA *vs*. AROS	POSA	24.7	29.6	27.8	19.8	21.6	30.2
	AROS	**75.3**	**70.4**	**72.2**	**80.2**	**78.4**	**69.8**

As in the randomly selected test locations, a descriptive analysis of the data from individual evaluation studies on these new locations suggests that AROS performs better than other approaches with larger mean values and narrower standard deviations. The mean and standard deviation of the scores obtained by the judges to PLACE are 2.97 ± 1.33 in comparison with AROS, 3.44 ± 1.19, while in the second study, these statistics obtained by POSA were 2.79 ± 1.25 in contrast with AROS, 3.5 ± 1.25. However, a Shapiro–Wilk test performed on these data shows that the score distributions also depart from normality with *p* < 0.01 in both studies, PLACE/AROS and POSA/AROS.

A chi-square test of homogeneity, with *α* = 0.05, was used to determine whether both score distributions were statistically similar on the data from the PLACE/AROS evaluation study, providing evidence that there is a difference in score distributions ( $\chi_{\left(4\right)}^{2}=35.92$ , *p* < 0.001) with a medium level of association (*V* = 0.192).

However, an omnibus *χ* ^2^ statistic does not provide information about the source of the difference between the score distributions. To this end, we performed a *post hoc* analysis following the standardized residuals method described in the work of Agresti (2018). As suggested by Beasley and Schumacker (1995), we corrected our significance level (*α* = 0.05) with the Sidak method (Šidák, 1967) to its adjusted version *α*_*adj*_ = 0.005, with critical value *z* = 2.81. The study revealed a significant difference in the qualification of the interactions generated by PLACE and AROS, with ours being qualified as natural more frequently.

The residuals associated with AROS indicate, with significant difference, that the interactions generated by our approach were marked as “not natural” less frequently than expected: *strongly disagree* (*z* = −4.4, *p* < 0.001) and *disagree* (*z* = −2.98, *p* = 0.002). Data also show a significant difference in favorable evaluations, where PLACE has less frequently positive evaluations than predicted by the hypothesis of independence in *agree* (*z* = −3.04, *p* < 0.001). We also observed a marginal significance, still in favor of AROS, in the frequency of *strongly agree* evaluations (*z* = −2.3, *p* = 0.015).

Not surprisingly, the chi-square test of homogeneity (*α* = 0.05) on the data from the POSA/AROS evaluation study revealed that there is strong evidence of a difference in score distributions ( $\chi_{\left(4\right)}^{2}=75.13$ , *p* < 0.001) with a larger level of association (*V* = 0.278). The *post hoc* analysis with standardized residuals concludes that the naturalness of human–scene interactions generated by AROS is, in the long term, better than that from POSA. Table 5 shows the cross-tabulated data of the scores observed by MTurkers and their standardized residual (critical value *z* = 2.81 for *α*_*adj*_ = 0.005).

**TABLE 5:** Cross-tabulation data of individual evaluation studies on challenging locations. A chi-square test of homogeneity on data provides evidence of difference in the distribution of scores with *α* = 0.05. An analysis of residual indicates the source of such differences, an asterisk (*) indicates conservative statistical significance at *α* = 0.05, and a double asterisk (**) denotes statistical significance with *α*_*adj*_ = 0.005. The best are in boldface.

Individual evaluation study	Model		1. Stronglydisagree	2. Disagree	3. Neither	4. Agree	5. Strongly agree
PLACE *vs*. AROS	PLACE	Observed frequency	81	131	54	161	59
		% within model	16.7%	27.0%	11.1%	33.1%	12.1%
		Standardized residual	4.44**	2.98**	−1.08	−3.04**	−2.43*
	AROS	Observed frequency	**36**	**92**	65	207	86
		% within model	**7.4%**	**18.9%**	13.4%	**42.6%**	**17.7%**
		Standardized residual	−4.44**	−2.98**	1.08	3.04**	2.43*
POSA *vs*. AROS	POSA	Observed frequency	86	141	93	122	44
		% within model	17.7%	29.0%	19.1%	25.1%	9.1%
		Standardized residual	4.95**	3.52**	1.70	−2.88	−6.57
	AROS	Observed frequency	**35**	**94**	73	**163**	**121**
		% within model	**7.2%**	**19.3%**	15.0%	**33.5%**	**24.9%**
		Standardized residual	−4.95**	−3.52**	−1.70	2.88**	6.57**

### 4.3 Qualitative results

Experiments verify that our approaches can realistically generate human bodies that interact within a given environment in a natural and physically plausible manner. AROS allows us to not only determine the location on the environment in which we want the interaction to happen (the where) but also select the specific type of interaction to be performed (the what).

**FIGURE 6:**
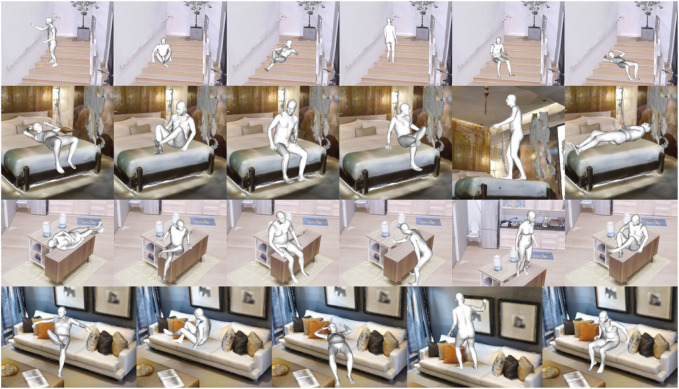
AROS shows good performance on a variety of novel scenes.

The number and variety of interactions detected by AROS can easily be increased as a result of its one-shot training capacity. The more trained the interactions, the more the human–scene configuration can detect/generate. Figure 6 shows examples of different affordance detections around single locations.

**FIGURE 7:**
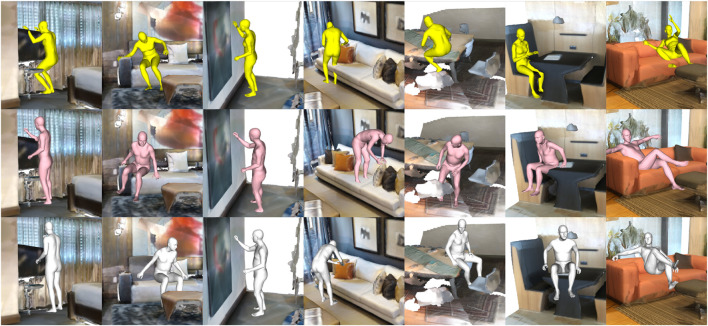
Qualitative challenging locations. PLACE (yellow), POSA (pink), and AROS (silver).

AROS showed better performance in more realistic environment configurations where elements, such as chairs, sofas, tables, and walls, are presented and must be considered during the generation of body interactions. Figure 7 shows some examples of interaction generated by AROS and baselines over challenging locations.

**FIGURE 8:**
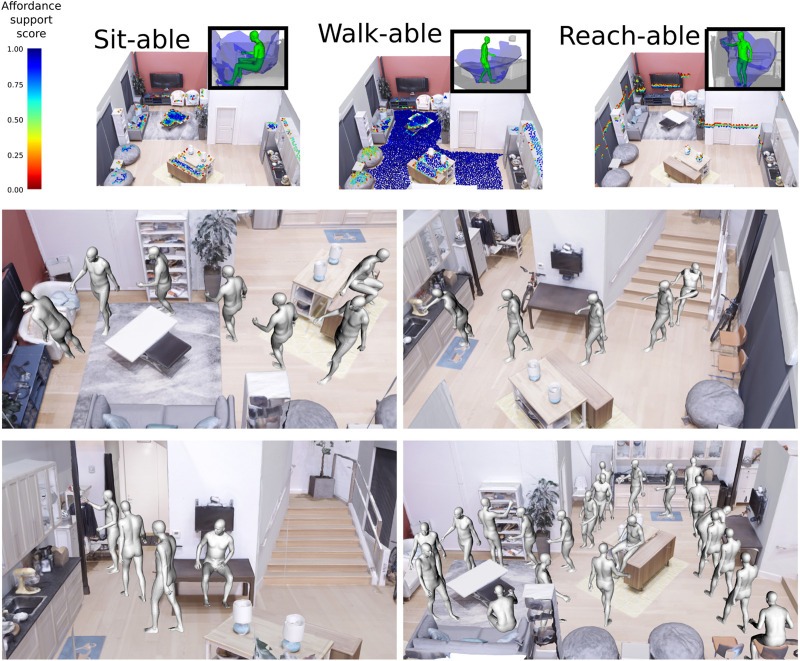
AROS can be used to create maps for action planning. Top: Many locations in an environment are evaluated for three different affordances (sit-able, walk-able, and reach-able). Bottom: AROS scores used to plan concatenated action milestones.

Alternatively, AROS can be used to concatenate affordances over several positions to generate useful affordance maps for action planners (see Figure 8). This can be used as a way to generate visualizations of action scripts or to plan the ergonomics and usability of spaces beyond individual objects.

## 5 Conclusion

In this work, we present AROS, a one-shot geometric-driven affordance descriptor that is built on the bisector surface and combines proximity zones and clearance space to improve the affordance characterization of human poses. We introduced a generative framework that poses 3D human bodies interacting within a 3D environment in a natural and physically plausible manner. AROS shows a good generalization in unseen novel scenes. Furthermore, adding a new interaction to AROS is straightforward, since it requires only one example. Via rigorous statistical analysis, results show that our one-shot approach outperforms data-intensive baselines, with human judges preferring AROS proposals 80% of the time over the baselines. AROS can be used to concatenate affordances over several positions. This can be used as a way to generate visualizations of action scripts in 3D scenes or to plan the ergonomics and usability of spaces beyond individual object affordances. We believe that explicit and interpretable description is valuable for complementing data-driven methods and opens avenues for further work, including combining the strengths of both approaches.

## Data Availability

The datasets presented in this study can be found in online repositories. The names of the repository/repositories and accession number(s) can be found at: https://abelpaor.github.io/AROS/.
