# Supplementary material for: AROS: Affordance Recognition with One-Shot Human Stances
Source: Front Robot AI. 2023 May 2;10:1076780. doi: 10.3389/frobt.2023.1076780 (PMC10185755; doi:10.3389/frobt.2023.1076780)
Supplement: Supplementary file 1 [file DataSheet1.PDF]

AROS: Affordance Recognition with One-Shot Human Stances

Supplementary material

1 TRAINING INTERACTIONS

We train 5 types of interactions: standing, walking, lying, reaching, and sitting (see Fig. S1). Training interactions were obtained from recordings in the PROX dataset Hassan et al. (2019). The data of each frame of the recordings include a 3D reconstruction of the observed scene, as well as an SMPLX representation Pavlakos et al. (2019) of the human interacting within the scene. For each pair (M_h, M_e), we also get its reference point p_{train} to calculate the AROS descriptor $(\mathcal{V}_{train}, \mathcal{C}_{train}, \hat{n}_{train})$. Training is thus based on the small selection of human-scene pairs per interaction. Note that semantic labels such as "sitting" are ambiguous, as there are various human poses that are considered sitting. But note that all of these are distinctive, e.g. sitting with trunk upright or sitting with trunk backwards and arms extended. In AROS, we use only one example to train each of these configurations.

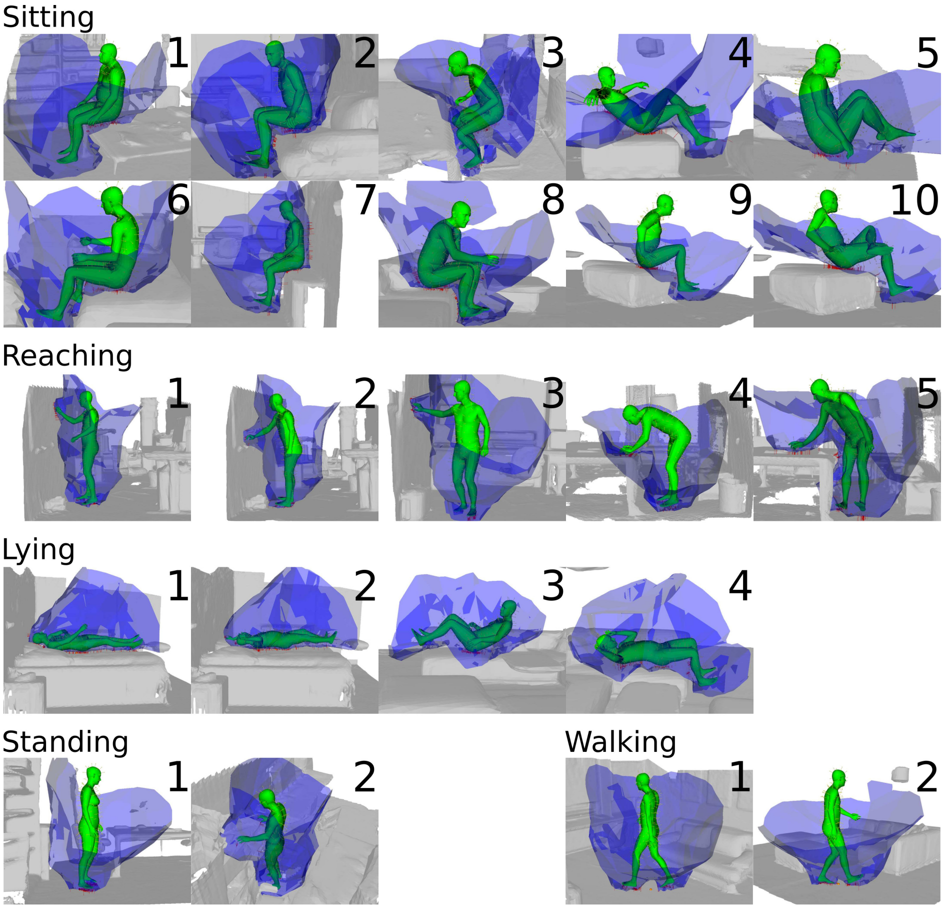

Figure S1. Interactions trained. 5 different categories of human-environment configurations. These are the 23 different interactions used for training in our experiments

2 SPHERICAL FILLERS

As described in the main text, we preprocess the noisy depth scans that are populated with missing scan regions (holes). We deal with this via the appending of spherical fillers. Spherical fillers with radius r are generated as follows: (1) Sample points from the environment mesh so that they are approximately evenly separated with a distance $6r/9$, (2) calculate the normal vectors of all sampled points, (3) keep samples from where a ray of size $2r$ can be propagated in the opposite direction to the normal without intersecting the environment mesh, (4) generate spheres tangent to the filtered sampled points, with centers in the rays directions, and (5) crop the spherical fillers with the environment mesh to avoid the creation of artifacts on the scanned scene.

The size and distribution of spherical fillers are dependent on the size and quality of the mesh. We empirically generate three sets of spherical fillers in all scenes from all three datasets as follows: The first, a set of spherical fillers of $r = 7[cm]$, aims to occupy the free space generated by large enough elements such as walls, floor, and sofas. The second set, spherical fillers with radius $r = 3[cm]$, is meant to fill small gaps of free space in the structure of the environment. The last set of spherical fillers with a radius of 12 [cm], generated on the extended bottom face (40 [cm]) of the scene bounding box, was designed to avoid misinterpretation of the boundaries of the scanned scenes. These values were used for all the dataset and provided here for repeatability. Some results are presented in Fig. S2.

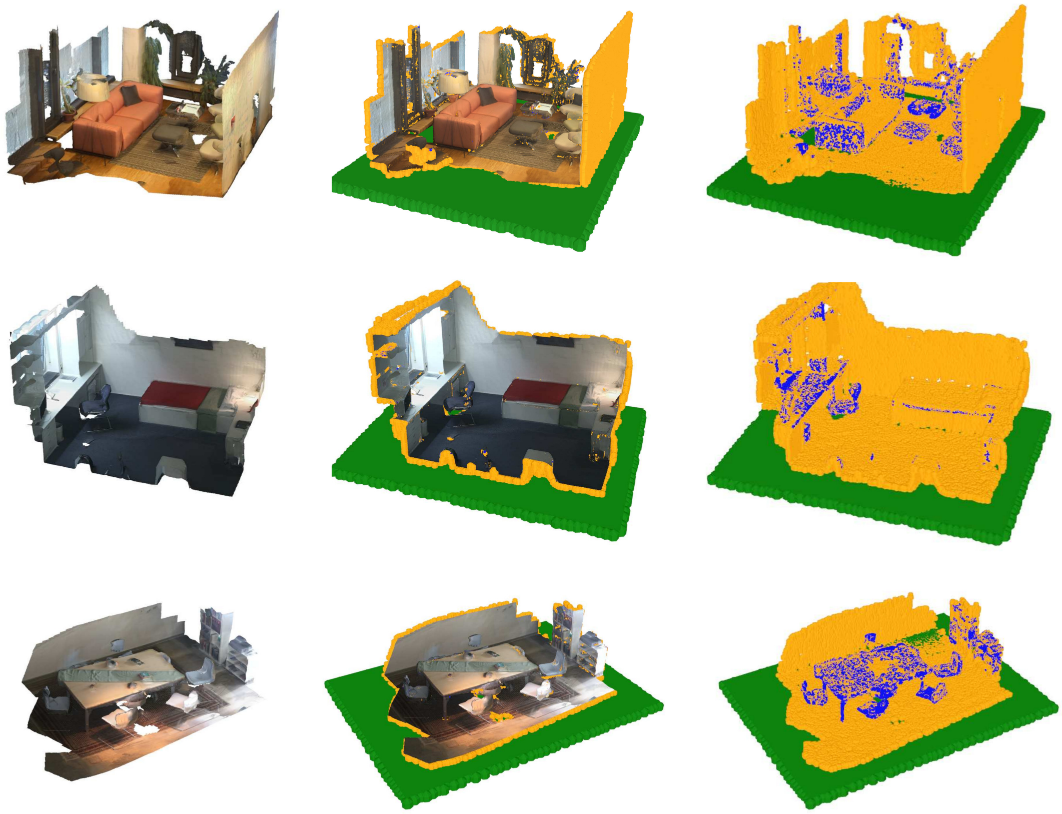

Figure S2. Examples of scene representations enhanced for better collision detection. From left to right: the original mesh definition of a scene, its enhanced version with sphere fillers, and the spherical fillers calculated. Large sphere fillers in green and yellow. Fine sphere fillers in blue

3 USER STUDIES

We designed and conducted two different protocols to evaluate the naturalness of generated human-environment pairs via Amazon Mechanical Turk. We used animated GIF images to present interactions to MTurkers. They observed them from different perspectives with a camera positioned to a moderated distance with respect the human body as shown in Fig. S3. In both protocols, every Mturker perform 11 evaluations per task with 2 control questions to remove unreliable assessors performing random answering. The control questions were selected to be as obvious as possible to be easy to decide for someone following the instructions. No MTurker was permitted to participate more than once.

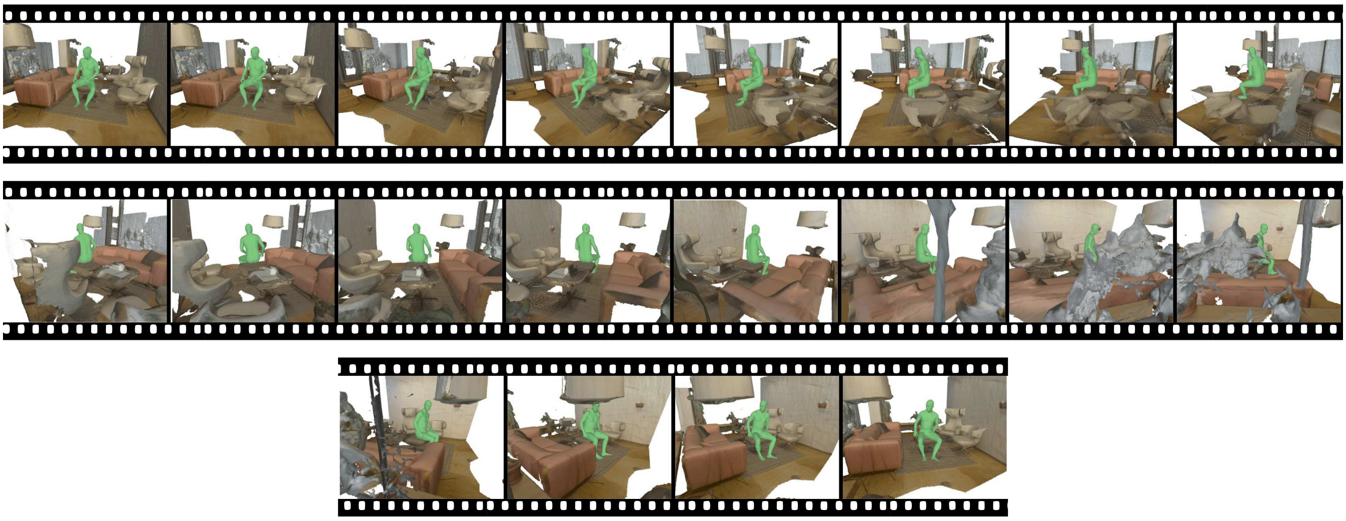

Figure S3. We use animated GIF images to visualize human-scene interactions for the MTurk studies

For the side by side evaluation study, body-environment pairs generated by both compared approaches Zhang et al. (2020) were presented to MTurkers for a direct comparison. To encourage a fair test, we ask both approaches to generate interaction around same scene locations with a tolerance of 1[m] around them. Left and right placement on the interface was randomized to prevent location bias. Our evaluation instrument, which includes instructions and the interface for MTurkers, is presented in Fig. S4.

For our individual evaluation study, only one interaction at a time per compared method was presented to an MTuker. The set of instructions given and the interface used to evaluate the interactions through a Likert scale is presented in Fig. S5.

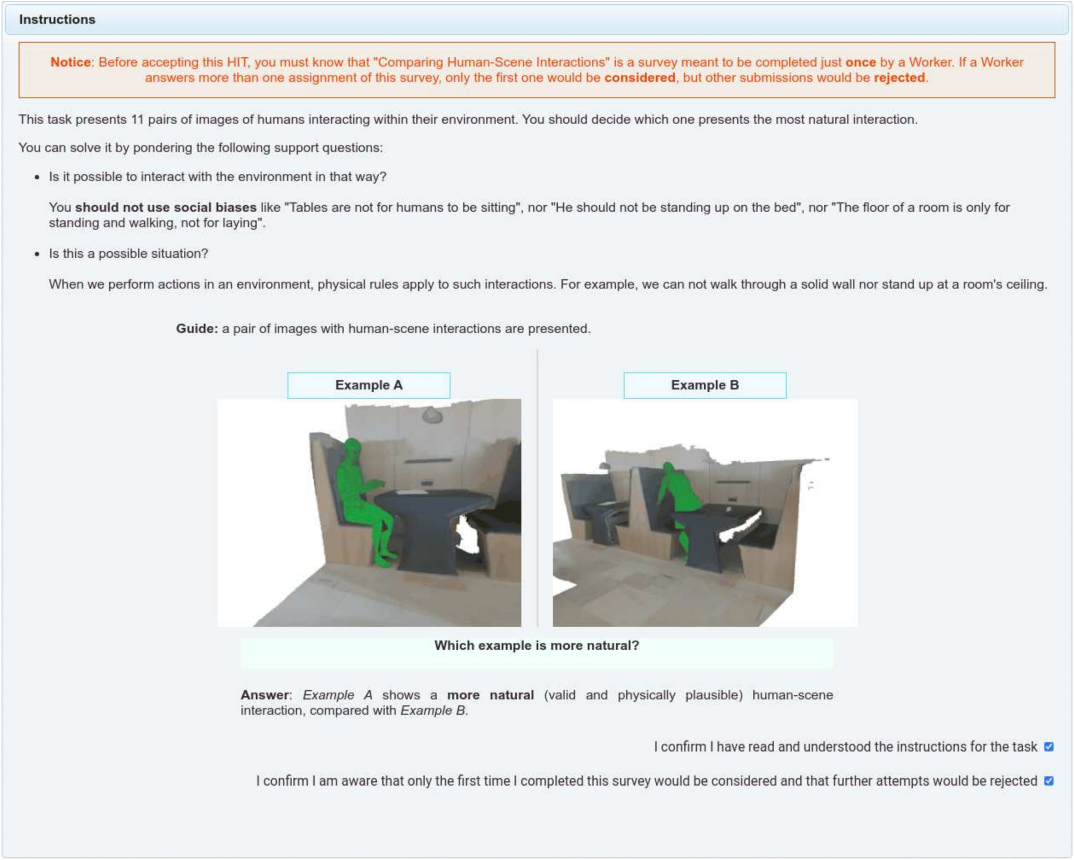

(a) Instructions for MTurkers

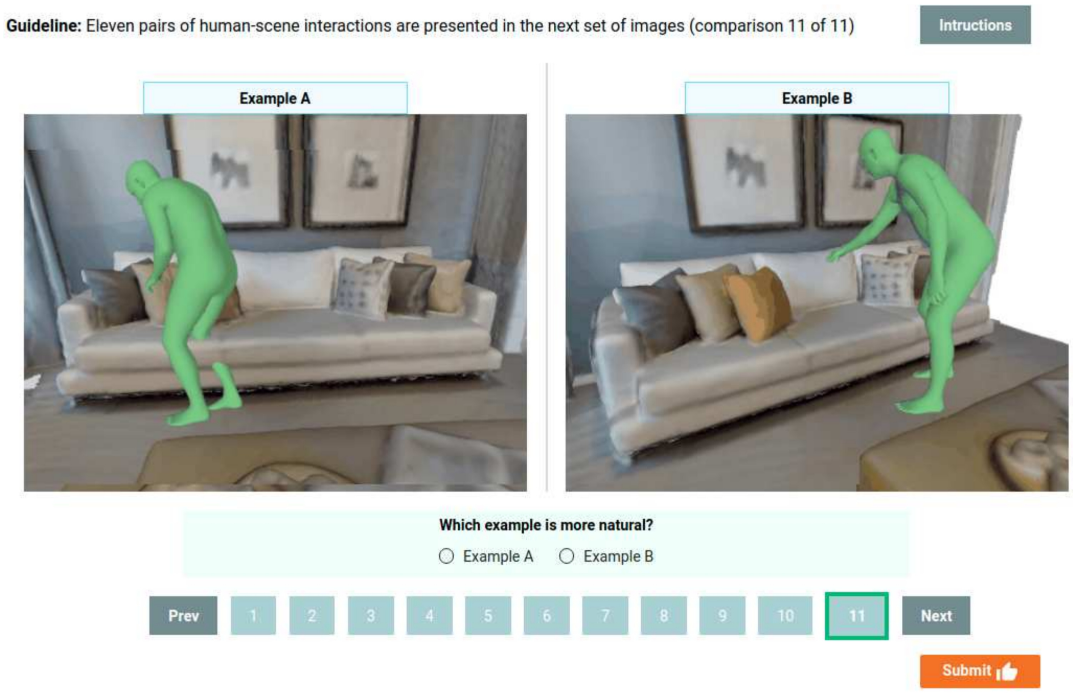

(b) Interface

Figure S4. Side by Side Evaluation Study

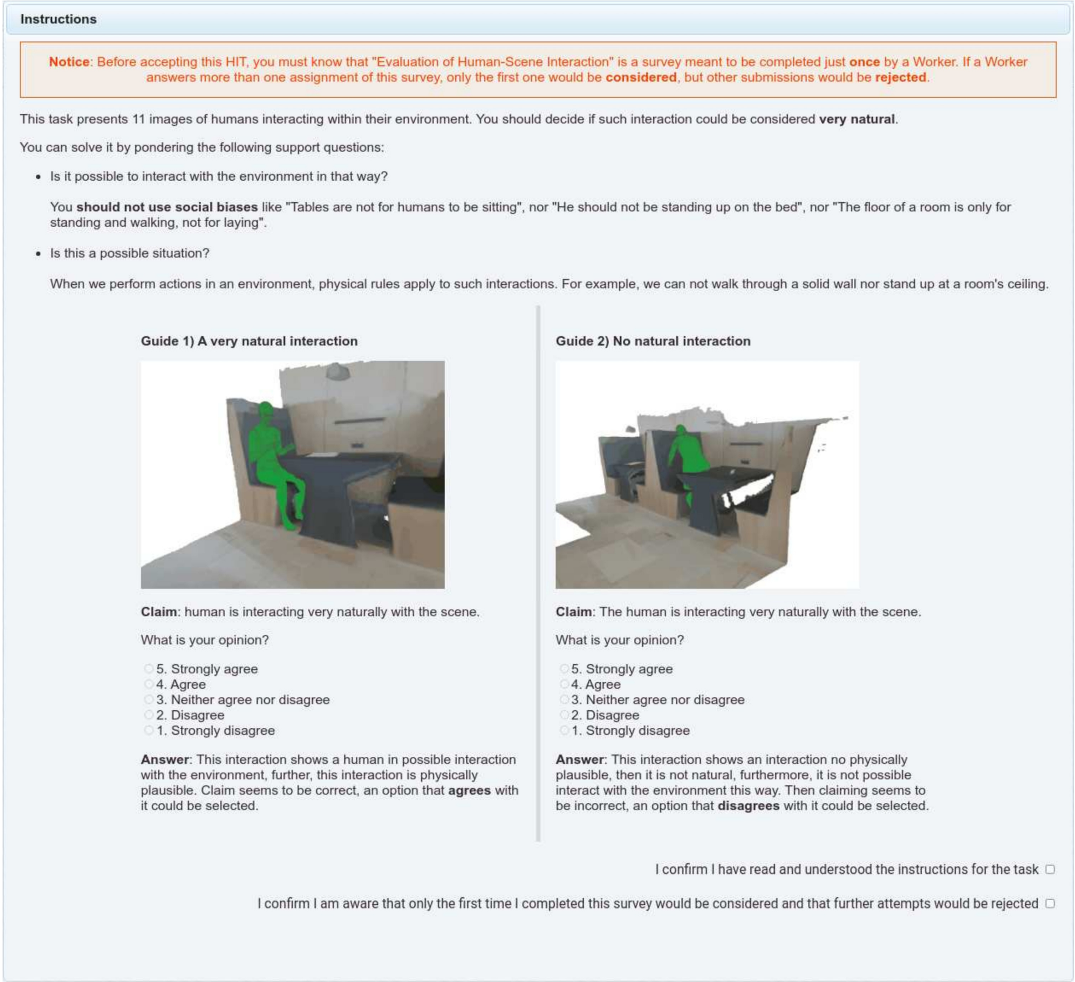

(a) Instructions for MTurkers

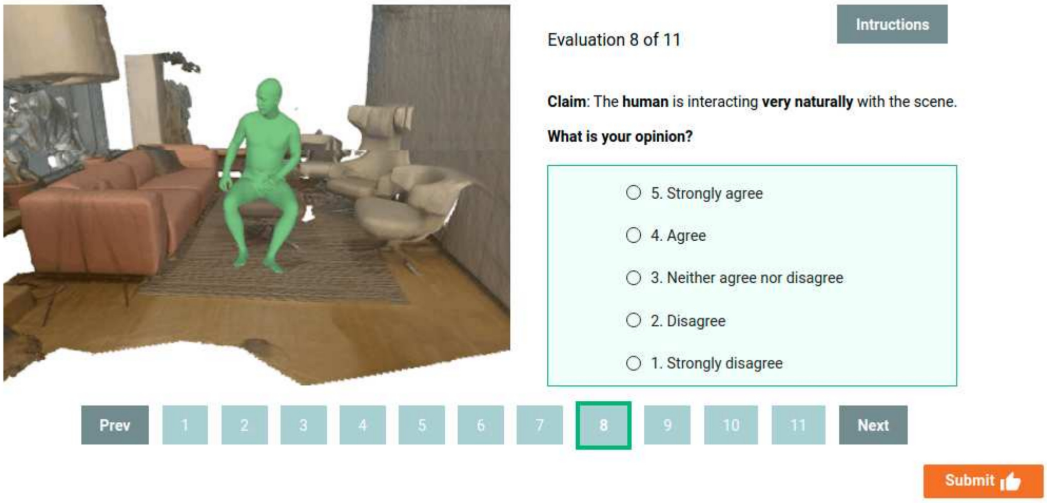

(b) Interface

Figure S5. Individual Evaluation Study

4 MORE ON QUALITATIVE RESULTS

AROS answers the question *what can a human perform here?* by detecting different affordances around the same location in an environment. Examples of this capability are presented in Fig. S6. More positive detections happened, but we presented only 4 in the figure for ease of legibility.

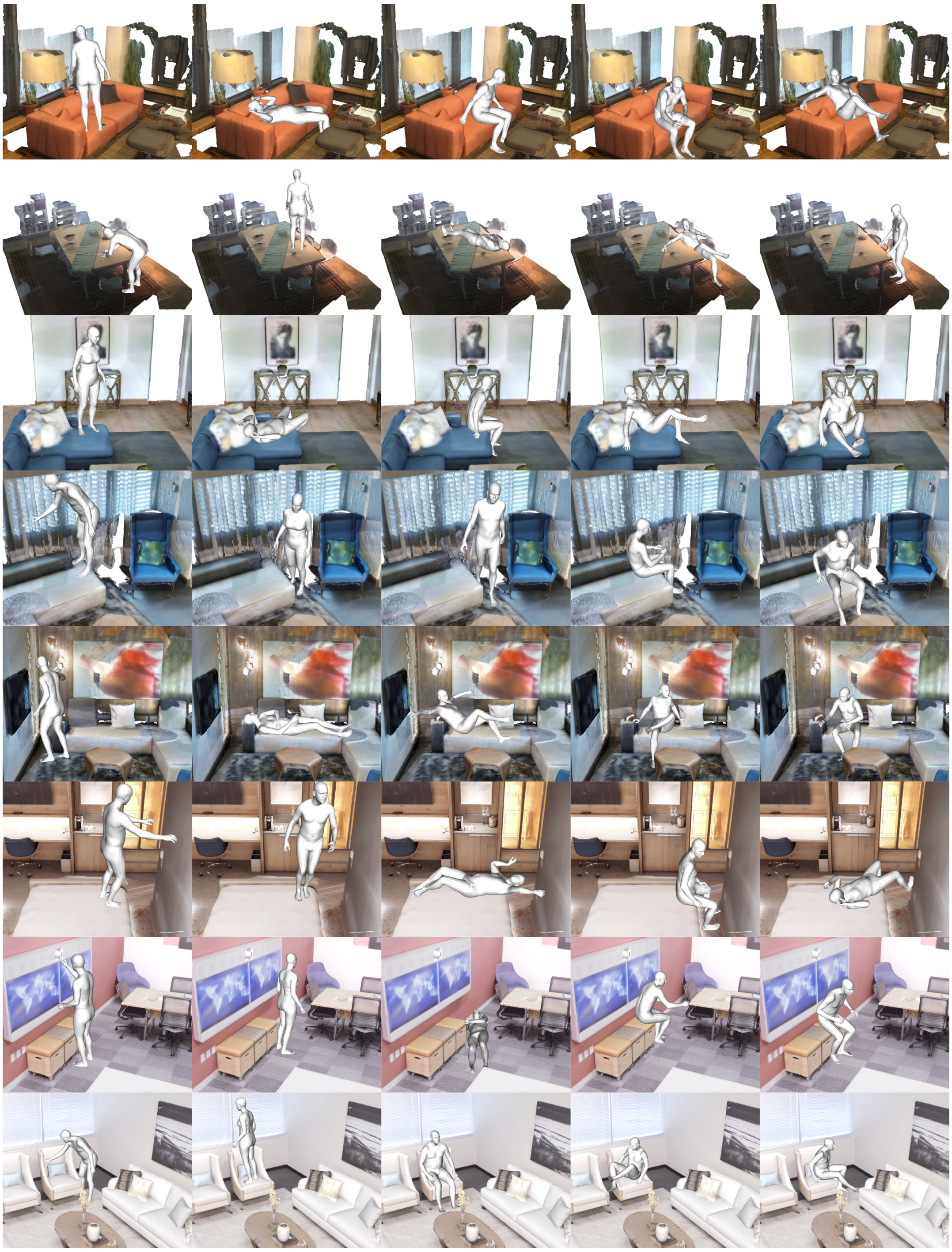

Figure S6. Further example human interactions generated by AROS. Several affordances are detected in the same location

Fig. S7 shows qualitative comparisons between PLACE (Zhang et al. (2020)), POSA (Hassan et al. (2021)), and our results. It is not possible to control the type of interaction we want PLACE to generate. In this set of examples, we asked PLACE to generate a human-environment configuration. Then, we classified the resulting interaction into one of the 5 affordances and requested POSA and AROS to detect and generate the same type of interaction.

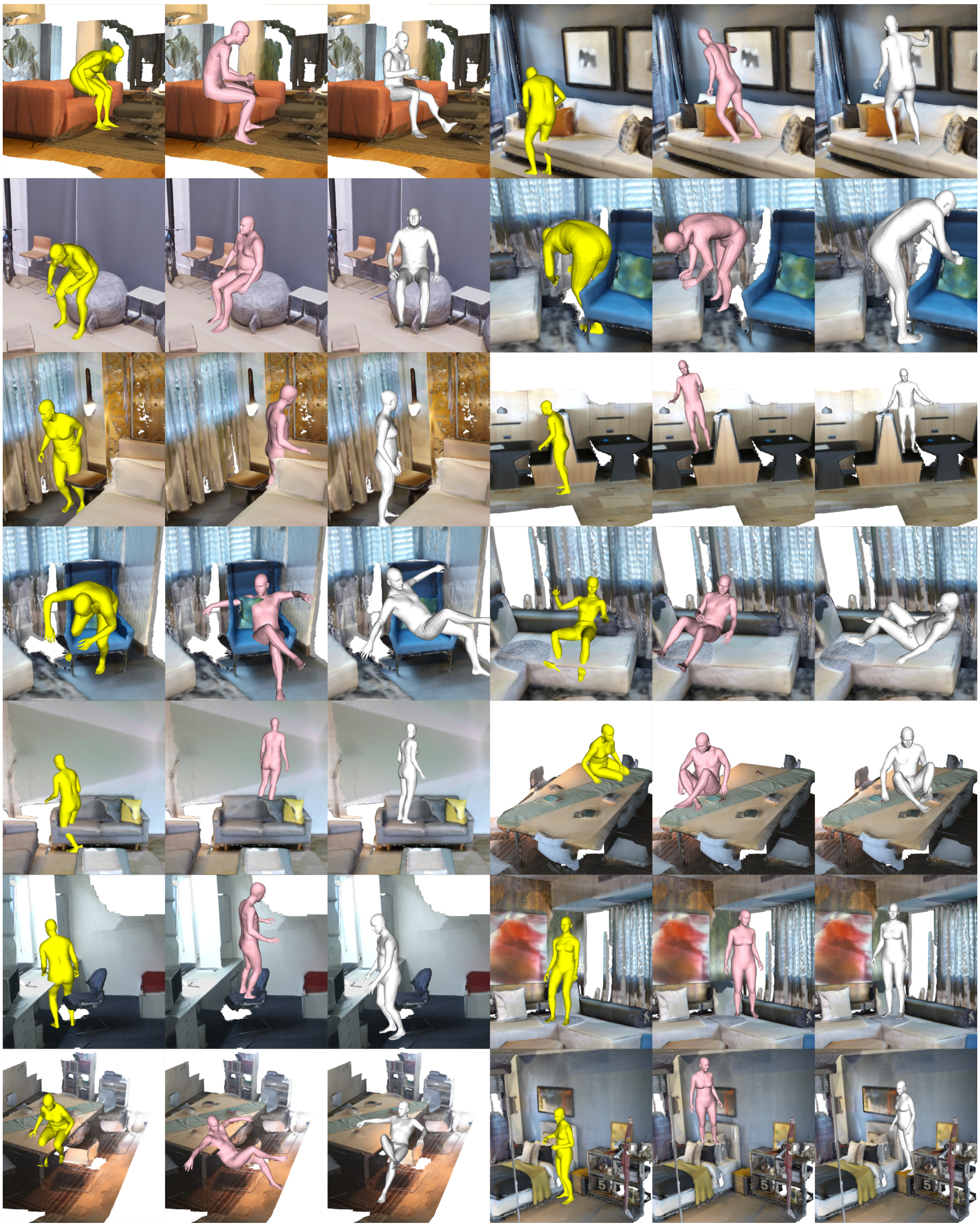

Figure S7. Human poses generated by PLACE (yellow), POSA (pink), and AROS (silver) on challenging locations in the scenes. AROS can often generate multiple and more plausible instances of the same affordance per location volume, here we show two examples

5 FAILURE CASES

Our evaluations show that bodies generated by AROS with AdvOptim optimization are natural and physically plausible. Nonetheless, it can still produce some failure cases with evident penetration in the environment surface.

There are two inter-related reasons for the failure cases. These are the density of *clearance vectors* and the density of the SDF values. Low densities in these parameters lead to being incapable of detecting a noticeable body-environment collision as they fail to properly characterize the space around thin structures in the scene. In our experiments, failures were mainly seen on tables when looking for positions capable of supporting a ”sitting” interaction, as shown in Fig. S8. Increasing densities for *clearance vectors* and/or the SDF will need to be balanced wrt compute time at inference.

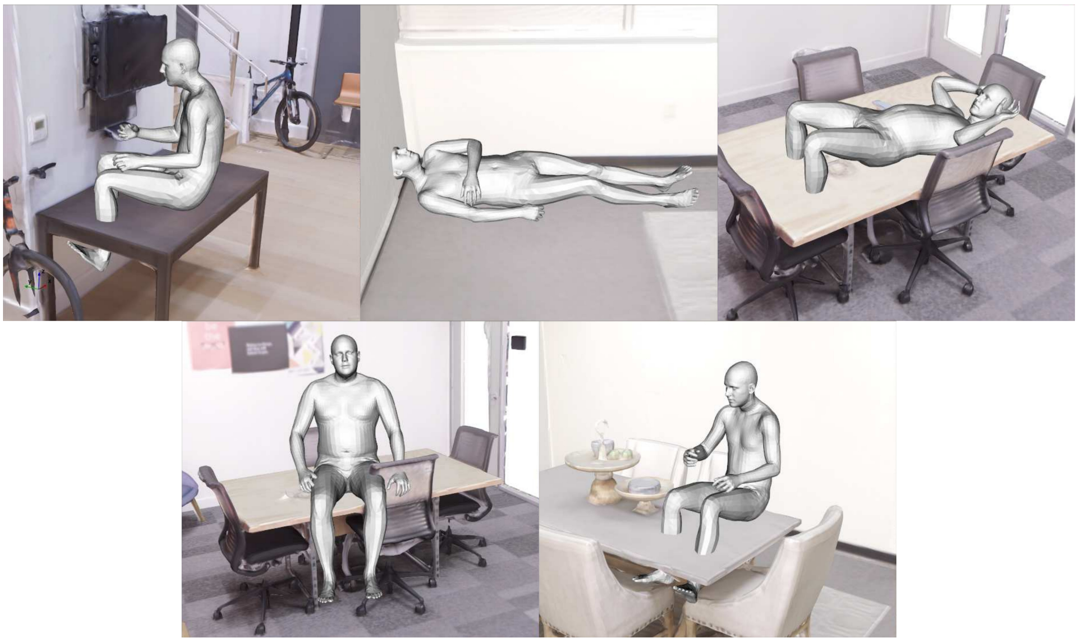

Figure S8. Examples of failure cases

6 EXECUTION TIME

The software implementation of AROS is available at https://abelpaor.github.io/AROS/. In our experiments, our approach evaluates the interaction support (affordance determination) of a location in an environment in 2.87 [s], while pose optimization lasts 17.94 [s] on average. AROS uses straightforward ray casting operations, so its computational complexity is O(log N).

REFERENCES

Hassan, M., Choutas, V., Tzionas, D., and Black, M. J. (2019). Resolving 3D human pose ambiguities with 3D scene constraints. In *Proceedings of the IEEE/CVF International Conference on Computer Vision*. 2282–2292

Hassan, M., Ghosh, P., Tesch, J., Tzionas, D., and Black, M. J. (2021). Populating 3D scenes by learning human-scene interaction. In *Proceedings of the IEEE/CVF Conference on Computer Vision and Pattern Recognition*. 14708–14718

Pavlakos, G., Choutas, V., Ghorbani, N., Bolkart, T., Osman, A. A. A., Tzionas, D., et al. (2019). Expressive body capture: 3d hands, face, and body from a single image. In *Proceedings of the IEEE/CVF Conference on Computer Vision and Pattern Recognition*. 10975–10985

Zhang, S., Zhang, Y., Ma, Q., Black, M. J., and Tang, S. (2020). PLACE: Proximity learning of articulation and contact in 3D environments. In *8th international conference on 3D Vision (3DV 2020)(virtual)*
